# Updates to the Current Landscape of Augmented Reality in Medicine

**DOI:** 10.7759/cureus.15054

**Published:** 2021-05-16

**Authors:** Sudarsan Murali, Kyle D Paul, Gerald McGwin, Brent A Ponce

**Affiliations:** 1 Department of Orthopaedic Surgery, University of Alabama at Birmingham (UAB) School of Medicine, Birmingham, USA; 2 Department of Epidemiology, University of Alabama at Birmingham (UAB) School of Medicine, Birmingham, USA; 3 Department of Orthopaedic Surgery, Hughston Clinic, Columbus, USA

**Keywords:** augmented reality, virtual reality, telementoring

## Abstract

Objective

With the introduction of the Google Glass in 2013, the use of augmented reality (AR) and virtual reality (VR) technology has been sharply accelerating in the field of medicine. Despite numerous hurdles and inadequacies identified with the initial devices, current product offering and the need for remote patient care has driven advancements and adoption of the newer generation of devices. This study aims to evaluate the current use of augmented reality devices and the current hurdles to implementation by surveying authors who have recently published on this topic.

Design

A 22-question survey was shared with authors of 27 recent publications relating to usage of augmented reality in medicine between the years of 2019 and 2020.

Results

Eighty-two percent of participants were located in North America while the rest were located in Europe. Interestingly, over 65% of respondents were over the age of 40. Almost half of respondents (45%) used the technology for image review while almost a third (27%) used it for capturing and sharing video. Most concerns to implementation were related to privacy (38%) or reimbursement (33%).

Conclusion

Despite the hurdles reported by respondents, the advancements in AR/VR have come a long way since their introduction and have great potential for continued usage in medicine. Despite this, however, it is important to recognize that cost, security, and battery life continue to serve as hurdles preventing the widespread adoption of this technology to mass markets.

## Introduction

With the introduction of the Google Glass in 2013, many advancements have occurred with the use and implementation of this and subsequent augmented reality (AR) and virtual reality (VR) technology in the field of medicine [1]. In 2016, a study of the feasibility and hurdles to implementation of this new technology was assessed, bringing up numerous issues relating to the software limitations, comfort, and patient data security of using the technology in medical applications [2]. Since then, many new iterations of both Google Glass and new competitors such as Microsoft’s HoloLens and Oculus Rift among others have re-shaped and re-defined the current technologic offerings [1]. With these new devices, not only can physicians record and visualize patient imaging as promised by previous devices, but they can also easily manipulate and interlay imaging studies to create a truly immersive and interactive virtual environment [3]. Furthermore, many of the concerns brought up in the previous study have been addressed, and there is a need to further assess and re-evaluate the current usage of and barriers to implementation of this technology. This study aims to better understand the current climate of AR and VR implementation in medical and surgical applications by surveying physicians who have published implementing this technology into their practices.

## Materials and methods

This survey was designed to rate and understand the current degree of usage of AR and VR in clinical applications while gathering similar data points as those evaluated in a previous study of this technology so that a comparison of previous and current use of AR/VR could be established [2]. A completely anonymous and de-identified survey was created and administered using the RedCap database (Vanderbilt University, Nashville, TN, USA) via an email using a uniform resource locator (URL). The email was addressed to a list of physicians who were the authors of 27 unique manuscripts published between 2019 and 2020 which were found using the keywords “augmented reality” and “virtual reality” on PubMed. This survey was determined to be exempt for review by the institutional review board.

The validated survey utilized in this study (see appendix) consisted of 22 questions in total. The first two collected general demographic data. The next 13 questions provided insight into the type of AR/VR technology used and characteristics of its use. The final seven questions assessed sentiment regarding hurdles to implementation or use. Percentages were calculated to evaluate frequency of responses.

## Results

Demographics

This survey was sent out four times during the survey period of two months via email to all physicians who were the authors of 27 recent publications in North America and Europe which were found using the keywords mentioned previously. Eleven experts with AR/VR experience completed the survey during the survey period. All physicians contacted in this survey were associated with an academic institution with 82% of respondents being located in North America and 18% were located in Western Europe. The breakdown of ages of responding physicians was as follows: 35% were under the age of 40 years, 27% were between 40 to 50 years, and 27% were over the age of 50 years.

Use characteristics

The most popular device used by the surveyed physicians was Microsoft’s HoloLens (45%), while the Google Glass, Epson Moverio and HTC Vive were tied for the second most popular (18%). Other less popular options included the Oculus Rift, Oculus Quest, Oculus Go, VR Surgical Simulator, and self-built simulators (9% each)(Table 1). All of the users utilized the technology in an academic hospital only. When asked about the reason for implementing AR/VR in their practice, 91% began using it due to clinical interest while 9% used it for simulation of clinical scenarios. Additionally, 18% started using AR/VR to increase efficiency, 27% wanted to extend the reach of healthcare, while 9% sought the ability to broadcast their work. Over half, 55%, used their devices during surgery while 27% used it in outpatient or inpatient setting while others used it for rounds, endoscopy, or image review. These devices were predominantly purchased using grants or departmental funding (55%). Nearly half, 45%, also used some institutional funding while 18% contributed out-of-pocket. An additional 18% of payments were made using industry relationships as well. Due to their use of their devices, 91% of physicians did not receive any additional reimbursement while 9% did receive some extra compensation from industry [4,5].

**Table 1:** Cost of Devices

Devices	Price (USD)
HoloLens	$3,500
Google Glass	$1,167
Epson Moverio	$699
HTC Vive	$695
Oculus Rift	$299

With regards to the functions accessed, 45% did image review or guidance and planning, while 27% did image/video capture or live transmission. Other uses included simulation, image segmentation, or pain management. All of our respondents have used their devices for over one year with 27% using it daily, 45% using it 1-5 times a month, 9% using it 5-10 times month, 9% using it 11-20 times a month, and 9% using it sporadically. More than half, 55%, of physicians found that their use of their devices has increased in their practice while 36% felt it has stayed the same or decreased (9%). Most physicians found the devices naturally intuitive (45%) while 36% found they needed some instruction, and 18% felt it was moderately difficult.

Device limitations

There was an even distribution of responses with an equal percentage that did not feel cost was prohibitive (14%) as there were respondents who felt the cost was prohibitive (14%). In contrast, respondents were more likely to feel that the cost of software associated with the device was not prohibitive (29%). Just over a quarter of respondents, 27%, had no issues with video and audio quality but the remaining felt that there were some prohibitive issues. Battery life was a prohibitive issue to 18%, but 27% did not experience any problems. In terms of function, 18% had no issues and another 18% only had mild problems, but the rest of the participants had issues ranging from moderately to extremely prohibitive. When ranking the most concerning aspects of AR/VR usage based on six options (Privacy/HIPPA, internet security, licensure/credentialing, reimbursement, potential malpractice, and patient acceptance), 38% felt privacy was the main concern while reimbursement (33%) and potential malpractice (22%) were the other top concerns. The least concerning aspect was also privacy for some respondents (37%) followed by reimbursement (33%) and licensure/credentialing (20%) (Table 2).

**Table 2:** Use Limitations AR: Augmented reality; VR: Virtual reality.

	No Issue	Mildly Prohibitive	Moderately Prohibitive	Prohibitive	Extremely Prohibitive
Cost					
Cost of AR/VR	14%	28%	14%	28%	14%
Software Cost	42%	14%	0%	28%	14%
Limitation					
Ergonomics	45%	9%	18%	27%	0%
Video/Audio	54%	9%	0%	27%	9%
Battery	54%	0%	18%	9%	18%
Overall Function	36%	9%	27%	9%	18%

## Discussion

With the current climate and state of medicine, there has been a quick adaptation to the use of remote and virtual-based mediums for medical care [6,7]. Especially with the strains forced upon the field of medicine as a result of the COVID-19 pandemic, many physicians and healthcare systems have felt increasing pressure to allow and encourage the implementation of both VR and AR systems to help protect and better serve patients and physicians [8,9]. As the technology currently stands, there have been multiple successful attempts at utilizing this technology to enhance communication, workflow, and the access to information for physicians at critical junctures during their treatment of patients [10,11]. Moving forwards, as the field of medicine slowly returns to the old normal, we anticipate this technology might have found a new place amongst the status quo and may continue to grow moving forwards. Though this current state has served as a driver for the development and use of AR/VR in practices, the improvements with the technology itself show a vast improvement from those evaluated previously [2].

Demographics

Similar to the previous study, it is no surprise that physicians who participated in the study were all affiliated with an academic institution. In contrast to 2016, however, we were surprised to see a drastic shift in demographics especially as it relates to age [2]. Compared to 80% of participants who were young physicians under the age of 40, this study saw a shift with only 35% of respondents being under the age of 40 and even 27% being over the age of 50 [2]. This change in age is a welcome alteration and provides some insight into the acceptance and interest of more established physicians and their desire to experiment and improve upon their current practice settings. Additionally, this also suggests greater acceptance by hospital leadership which is frequently made up of more senior physicians. In contrast to the previous study, this age gap was seen as a hurdle to overcome by the Google Glasses, but seemingly one that has seen modest improvements since the introduction of AR/VR technology to the medical field [2]. With over 45% of respondents stating that their devices were extremely easy to learn and use, it seems that device manufacturers have created a user-friendly solution to their predecessors.

Use characteristics

In terms of device selection and use, there is an apparent clear winner in this study with almost half of users choosing to work with Microsoft’s HoloLens (45%) over fellow competitors. With the multiple options available today, it is interesting to see one device hold such a lead over other competitors such as Google Glass which was surveyed previously and has been around longer. Similar to the previous study, a majority of users were drawn to this technology out of clinical interest and curiosity (91%) [2]. With this study, we also saw changes suggesting more consistent use of devices in their practice with all respondents using their device for over one year and 27% using their device daily. In fact, over 55% of respondents shared that their use of AR/VR has been increasing in their current practice. This trend is further evidenced by the recent uptrend in publications containing keywords such as “augmented reality” with almost 700 manuscripts being published in 2020 compared to just 270 in 2017. With the growing interest and support for this technology, this makes it an interesting juncture to re-evaluate the current climate and acceptance of these devices as they begin to gather support within multiple fields of medicine.

In this study, physicians reported using their devices in many different settings ranging from surgical cases to outpatient clinic visits, aligning with the growing body of literature of different applications of AR/VR in the field of medicine [10-13]. In contrast to the results of the previous study where users primarily used their devices for hands on procedures, we see that there has been a rapid expansion to apply this technology to multiple settings. Despite these changes, most physicians in this study did not receive additional compensation for their services rendered using their AR/VR device. Though this does not seem to be an issue, it may continue to serve as a barrier to entry for many with over 50% of respondents relying on departmental funds to acquire their AR/VR headset for patient care. Despite this, it is interesting to see so many physicians continue to use their devices regularly, further emphasizing the value and convenience these devices provide to the physicians.

Device limitations

Despite the values provided to physicians, the cost continues to pose a hurdle for many physicians with only 14% reporting that cost was not an issue. Though this is not a new issue with these devices, it is a point of concern with the lack of increased compensation as described above. Though many improvements have been made to these devices since our previous study, some issues like battery life (3-4 hours) do continue to pose a problem for many users [2]. In some studies, many users experienced battery failure in the middle of procedures suggesting that this is a primary hurdle that remains to be addressed by device manufacturers [11,14]. Despite this, other studies and our respondents do continue to show the vast improvement of the device and its capabilities in recent devices [14]. Other key improvements have also been noted as it relates to information security and HIPAA concerns over data used on devices. In our previous survey, over 60% felt that information security was a major prohibitive factor to their use of technology, however only 38% of this study’s respondents felt it was prohibitive [2]. There is still clearly work to be done to gain approval by all physicians and to ensure information security, but it is evident that progress has been made to help make this technology more widely accessible.

Limitations

There are multiple limitations to this study. To gather participants, this study relied on the accuracy of co-authors listed on recent publications fitting keywords used to identify relevant publications since no other database of device users exist. As such, we may not have gotten an accurate perspective of the greater population of physicians utilizing AR/VR devices. Additionally, this study had a low number of participants (11), but being a niche topic, we feel that the experiences of these published experts do provide a valuable yet likely narrow perspective. To address these, further studies are warranted to gather a more accurate representation of physicians’ use of this technology.

## Conclusions

With the current iteration of these AR/VR devices, it is clear that major strides have taken place since the previous survey of this technology. With these perspectives and current literature in this area, it is evident that AR/VR technology usage and acceptance is on the rise in multiple fields of medicine. Despite this, however, it is important to recognize that cost, security, and battery life continue to serve as hurdles preventing the widespread adoption of this technology to mass markets. It is encouraging to see that many of the technological concerns have been addressed, and this suggests that device makers are working to create a more suitable product for medical use. With the momentum gathered by the necessity of such technology during the pandemic, we do feel that this technology is likely to continue its trajectory to become a more widely used and accepted vehicle for the delivery of care and as a tool for physicians in a variety of settings.

## Appendices

Demographics

Please specify your region.

North America

South America

Western Europe

Eastern Europe

Middle East

South Asia

East Asia

Australia

Africa

Other (please specify)

Age

25-29

30-35

36-40

41-45

46-50

Over 50

Technology Usage

Which form of AR/VR technology do you use?

HoloLens

Google Glass

Oculus Rift

HTC Vive

Other?

In what setting did you use AR/VR technology?

Academic hospital

Private hospital

Government hospital

Military hospital

Outpatient surgery center

Outpatient clinic

Other (please specify)

What reason led you to your eventual clinical application of AR/VR?

Clinical interest

Monetary concerns

Fewer administrative regulations

Fewer tech restraints (power, wifi access, etc.)

COVID-19 

Other (please specify)

What was the clinical situation where you used AR/VR? (Choose all that apply)

Surgery

Clinic

Outpatient Care

Inpatient Care

Patient Rounds

Other (please specify)

How was the clinical situation where you used AR/VR paid for? (Choose all that apply)

Grant

Personal

Departmental

Institutional/Hospital

Industry

Other (please specify)

Have you received any additional compensation beyond routine clinical reimbursement for services rendered while using AR/VR?

Yes

No

Who provided the additional reimbursement?

Patient

Hospital

Industry

Insurance Company

Medicare

Medicaid

Other (please specify)

What functions have you accessed when using AR/VR? (Choose all that apply)

Medical records review

Imaging review

Vitals monitoring

Guidance/planning

Video recording

Live encounter transmission

Other (please specify)

How long have you used AR/VR technology?

Less than 1 week

1 week-1 month

1 month to 6 months

6 months to 1 year

Over 1 year

How difficult was it to learn how to use AR/VR?

Naturally intuitive (no instruction of any kind necessary)

Easy but required instruction

Moderately difficult

Difficult

What made you seek the use of your current AR/VR device? (Choose all that apply)

Personal archive

Ability to broadcast

General interest in technology

Marketing/Public Relations

Increase efficiency

Extend reach of healthcare

Dissatisfaction with other recording or video devices

Other (please specify)

How often do you use AR/VR?

Daily

1-5× per month

5-10× per month

11-20× per month

21-29× per month

Only used on a few isolated occasions

The use of AR/VR in your clinical practice has:

Increased

Remained the same

Decreased

Stopped

Cost

Rate the following based on the degree to which each was a potential obstacle to using AR/VR at your institution

Cost of AR/VR and Any Associated Systems.

No issue

Mildly prohibitive

Moderately prohibitive

Prohibitive

Extremely Prohibitive

Software Cost

No issue

Mildly prohibitive

Moderately prohibitive

Prohibitive

Extremely Prohibitive

Limitations

Rate the following based on the degree to which each was a potential obstacle to using AR/VR at your institution

Ergonomics (comfort, positioning, weight, etc.)

No issue

Mildly prohibitive

Moderately prohibitive

Prohibitive

Extremely Prohibitive

Video/Audio Quality

No issue

Mildly prohibitive

Moderately prohibitive

Prohibitive

Extremely Prohibitive

Battery Life

No issue

Mildly prohibitive

Moderately prohibitive

Prohibitive

Extremely Prohibitive

Overall Functionality

No issue

Mildly prohibitive

Moderately prohibitive

Prohibitive

Extremely Prohibitive

Risk

Please rank with #1 being the most concerning and #6 being the least concerning in your decision to use AR/VR.

Patient Privacy Concerns/Regulations (HIPAA)

Internet Security

Licensure/Credentialing

Reimbursement

Potential Malpractice

Patient Acceptance
